# Supplementary material for: Chitin is a functional component of the larval adhesive of barnacles
Source: Commun Biol. 2020 Jan 17;3:31. doi: 10.1038/s42003-020-0751-5 (PMC6969031; doi:10.1038/s42003-020-0751-5)
Supplement: Supplementary file 1 — Supplementary Information [file 42003_2020_751_MOESM1_ESM.pdf]

(A)

>Megabalanus_rosa_ Mr-lcp1-122k

RVLLLLPLALAAAVLSVSATKYVYPRAISHRRPVRYLIQRSANPRIAFSFIEAQLCQPLM
RQLLRSRYPRPTPELLRQLTSSRRQLPPVKQLQTLLNGLYRQRVGPIYDGLLPYVMYTRA
GRLFSRKSLLNPLTRLSASKIQLQFLKFLTRIDPRLMKENIGDNIFSEVRGSLAGINRSY
RRLSVPVLRNLYNTFVSVIGGNGRSQFLLGLSQRQRLQHFSTSLGLYLGQNRNQLPTLLK
NNALMYQDFAGDIDGQYILHSSGIRLLGQLQQQQQSEADRFFHQLEHDHFEAVSDVLSSI
FRVQRSISWKSLPHLGYSGLLARRFYPGLDVGQTDIIARYILSALSGYGAVSRSAFVKTG
GLVNSLQYLPYVRGLPSNTVSTIQQADLARILGGLNLPGLSPRLTPGILQAARRSQFITQ
VMLAALMHGVRFTTPQQSTLLFRDVFASLLMQEKNFNAARFTSFLGDRLRPFALMCRVTI
PNMRPAIRRIPDIRMLPLSGIISLQDALRRGLIQLPAGLARRVGIIVRPRRHVIPVQPPA
PTEPPFPSIVVSRITLRSVVARRVYVLLHGSIPILTVEMLQPVLQLLVEREVVPVVLLRS
PDKLVVRLQQVLKAVRPLLVMATKAAMVPSFVTDKAFTSDIARVAFILDILVIYAHGYTE
LQPPLVTNSVQAILRAYYGANKAFQPVPYLQYSNQYFPQLVARGLLARPLRLGSMTLPAL
TVTNTLSGLRPVYGRVTYHGLGSVLSYPGAAPLLAGIKGTDSAALIRALTFGATKPVFQL
SQGDVSGLQDILKPLTLFDKKYSVQDLQFLFAFGVRDLRIPRSIYSSRITSLFRESLVSF
IGTKKPLITNRIYNPAYLNLVINDFLVDKLTSGYLSPRVQVRVPAGQSVTLRRAQLTSLM
RQSLQPLRLRMLPSYLGQLLTLAIPEVRVLRTVKPTSLLPSFGRYLGSGPFRNFRFSLTD
NEFSGILGQLRSQRYLPGFFLRYPPRLQRYYFDNLFGSFAAYSLVRQSSEQFSGGALRHS
VVYNSFSRSLIPSFFRSRKFTNLGDLFGGAPDYFSLCRRTFRTLPGLQIIRHPSNVGGIH
HVVNGRMVGSYKAAKASGFFSK

>B_amphitrite_k55_2957215

LVQPIYPGLLPYYLYNRAGSRLFSKKSLLSPLRRLTPSDVQLQFLRFLTRIDRRLVSEPV
GDDVFSAVRQSLVDVNSGYGGLSVPLLRNLYGTFVSVIGGNGRSRFLLGLSPQRRLQHFS
TSLGLYLRAARGQLPALLKNSALLYQDFAGDVDSRYILHSSGIRLLGELERQQESEADRF
FLELEHDHFEAVNDVLSSIFRVQRSLSLSSLPRLGYSGVLARRFYPGLDVAQTDVIARYI
LRALSSYGAISGSVAKTGGLLQSLPYLPYVRGLPVNTVSSIQQADLASILGSLNLSGLRS
RLTPEVLQAARGGPFITQALLASLLHGVRFTSPQQGVLLFRDLLASLLMHEKRFDPAQFT
SFLGDPIRPFVLLNRIIVPNTLPAIRRIPKIQLLPLNSILSLRGALQRGIVQLPPG

>B_amphitrite_k75_2477949

PSIVVSRVSIGSAAARRLFALLSGGIPVLTVGLLQPVLQLLVERQVVPVALLRAPDQLEA
RLQDLLKALQPLVSVASQAATVPSFVTDKTFTADSARVAYILDLLVIYSRGYTVLEAPLI
SSSVIATLRSYYTANRAFEPIPFLAYSDRFFPQLIAQGLLARPLRLGSSTLSPATVTATL
AGLRPAYGPVTYHGLASVLGYRGAAPLLRGVDLTDPTALVRX

>B_amphitrite_k75_2510032

LRPAYGPVTYHGLASVLGYRGAAPLLSGLDLTDPKALVRALTFGASKPAFQLSESEVSGL
QEILKPITLFDRQYSLADLQFLFSFGVRELRIPRSIYGGRLAPLFRESLTSFIATRKSLI
ADRAYNPAYLRLVVDQLLVDKLTSGELSPRVQVRIPSGRLTLRRSQVSRLVRRSLLP

>B_amphitrite_k55_979678

LRRSQVSRLVRRSLLPLQLRLLPPFVGQLLTVAVPEVRLLRHAKPASLLSSFGQYLGSEP
FRRFRFSLADDEFSAVLGALTRQRRLPGFFLRYPSRLQRYYLDNLFGSFAAYSLLRYNAQ
ELVTEPLSHSALFESFSTSLVPSFFKGHKFSGLGDLLGVAPDYFTLCRRTFSTLPGLQAI
RPPVHIAX

(B)

> Megabalanus_rosa_ Mr-lcp1-122k

RVLLLLPLALAAAVLSVSATKYVYPRAISHRRPVRYLIQRSANPRIAFSFIEAQLCQPLM
RQLLRSRYPRPTPELLRQLTSSRRQLPPVKQLQTLLNGLYRQRVGPIYDGLLPYVMYTRA
GRLFSRKSLLNPLTRLSASKIQLQFLKFLTRIDPRLMKENIGDNIFSEVRGSLAGINRSY
RRLSVPVLRNLYNTFVSVIGGNGRSQFLLGLSQRQRLQHFSTSLGLYLGQNRNQLPTLLK
NNALMYQDFAGDIDGQYILHSSGIRLLGQLQQQQQSEADRFFHQLEHDHFEAVSDVLSSI
FRVQRSISWKSLPHLGYSGLLARRFYPGLDVGQTDIIARYILSALSGYGAVSRSAFVKTG
GLVNSLQYLPYVRGLPSNTVSTIQQADLARILGGLNLPGLSPRLTPGILQAARRSQFITQ
VMLAALMHGVRFTTPQQSTLLFRDVFASLLMQEKNFNAARFTSFLGDRLRPFALMCRVTI
PNMRPAIRRIPDIRMLPLSGIISLQDALRRGLIQLPAGLARRVGIIVRPRRHVIPVQPPA
PTEPPFPSIVVSRITLRSVVARRVYVLLHGSIPILTVEMLQPVLQLLVEREVVPVVLLRS
PDKLVVRLQQVLKAVRPLLVMATKAAMVPSFVTDKAFTSDIARVAFILDILVIYAHGYTE
LQPPLVTNSVQAILRAYYGANKAFQPVPYLQYSNQYFPQLVARGLLARPLRLGSMTLPAL
TVTNTLSGLRPVYGRVTYHGLGSVLSYPGAAPLLAGIKGTDSAALIRALTFGATKPVFQL
SQGDVSGLQDILKPLTLFDKKYSVQDLQFLFAFGVRDLRIPRSIYSSRITSLFRESLVSF
IGTKKPLITNRIYNPAYLNLVINDFLVDKLTSGYLSPRVQVRVPAGQSVTLRRAQLTSLM
RQSLQPLRLRMLPSYLGQLLTLAIPEVRVLRTVKPTSLLPSFGRYLGSGPFRNFRFSLTD
NEFSGILGQLRSQRYLPGFFLRYPPRLQRYYFDNLFGSFAAYSLVRQSSEQFSGGALRHS
VVYNSFSRSLIPSFFRSRKFTNLGDLFGGAPDYFSLCRRTFRTLPGLQIIRHPSNVGGIH
HVVNGRMVGSYKAAKASGFFSK

>B_amphitrite_k55_2957215

LVQPIYPGLLPYYLYNRAGSRLFSKKSLLSPLRRLTPSDVQLQFLRFLTRIDRRLVSEPV
GDDVFSAVRQSLVDVNSGYGGLSVPLLRNLYGTFVSVIGGNGRSRFLLGLSPQRRLQHFS
TSLGLYLRAARGQLPALLKNSALLYQDFAGDVDSRYILHSSGIRLLGELERQQESEADRF
FLELEHDHFEAVNDVLSSIFRVQRSLSLSSLPRLGYSGVLARRFYPGLDVAQTDVIARYI
LRALSSYGAISGSVAKTGGLLQSLPYLPYVRGLPVNTVSSIQQADLASILGSLNLSGLRS
RLTPEVLQAARGGPFITQALLASLLHGVRFTSPQQGVLLFRDLLASLLMHEKRFDPAQFT
SFLGDPIRPFVLLNRIIVPNTLPAIRRIPKIQLLPLNSILSLRGALQRGIVQLPPG

59% coverage
72% identity

>B_amphitrite_k75_2477949

PSIVVSRVSIGSAAARRLFALLSGGIPVLTVGLLQPVLQLLVERQVVPVALLRAPDQLEA
RLQDLLKALQPLVSVASQAATVPSFVTDKTFTADSARVAYILDLLVIYSRGYTVLEAPLI
SSSVIATLRSYYTANRAFEPIPFLAYSDRFFPQLIAQGLLARPLRLGSSTLSPATVTATL
AGLRPAYGPVTYHGLASVLGYRGAAPLLRGVDLTDPTALVRX

34% coverage
65% identity

>B_amphitrite_k75_2510032

LRPAYGPVTYHGLASVLGYRGAAPLLSGLDLTDPKALVRALTFGASKPAFQLSESEVSGL
QEILKPITLFDRQYSLADLQFLFSFGVRELRIPRSIYGGRLAPLFRESLTSFIATRKSLI
ADRAYNPAYLRLVVDQLLVDKLTSGELSPRVQVRIPSGRLTLRRSQVSRLVRRSLLP

29% coverage
66% identity

20% coverage
69% identity

>B_amphitrite_k55_979678

LRRSQVSRLVRRSLLPLQLRLLPPFVGQLLTVAVPEVRLLRHAKPASLLSSFGQYLGSEP
FRRFRFSLADDEFSAVLGALTRQRRLPGFFLRYPSRLQRYYLDNLFGSFAAYSLLRYNAQ
ELVTEPLSHSALFESFSTSLVPSFFKGHKFSGLGDLLGVAPDYFTLCRRTFSTLPGLQAI
RPPVHIAX

Supplementary Figure 1*:* Identification of a cement gland-specific Mr-lcp1-122k by MS/MS, and alignment to equivalent sequences in the B. amphitrite transcriptome. **A)** MS/MS-generated peptides mapped onto (left) the *M. rosa* full-length sequence and (right) four contigs from the *B. amphitrite* transcriptome. **B)** Mapping of the *B. amphitrite* sequences onto the *M. rosa* sequence. Sequences sharing significant identity are indicated by their colour. The coverage of the respective *B. amphitrite* sequence and its identity match to the equivalent stretch of *M. rosa* sequence are indicated for each *B. amphitrite* contig.

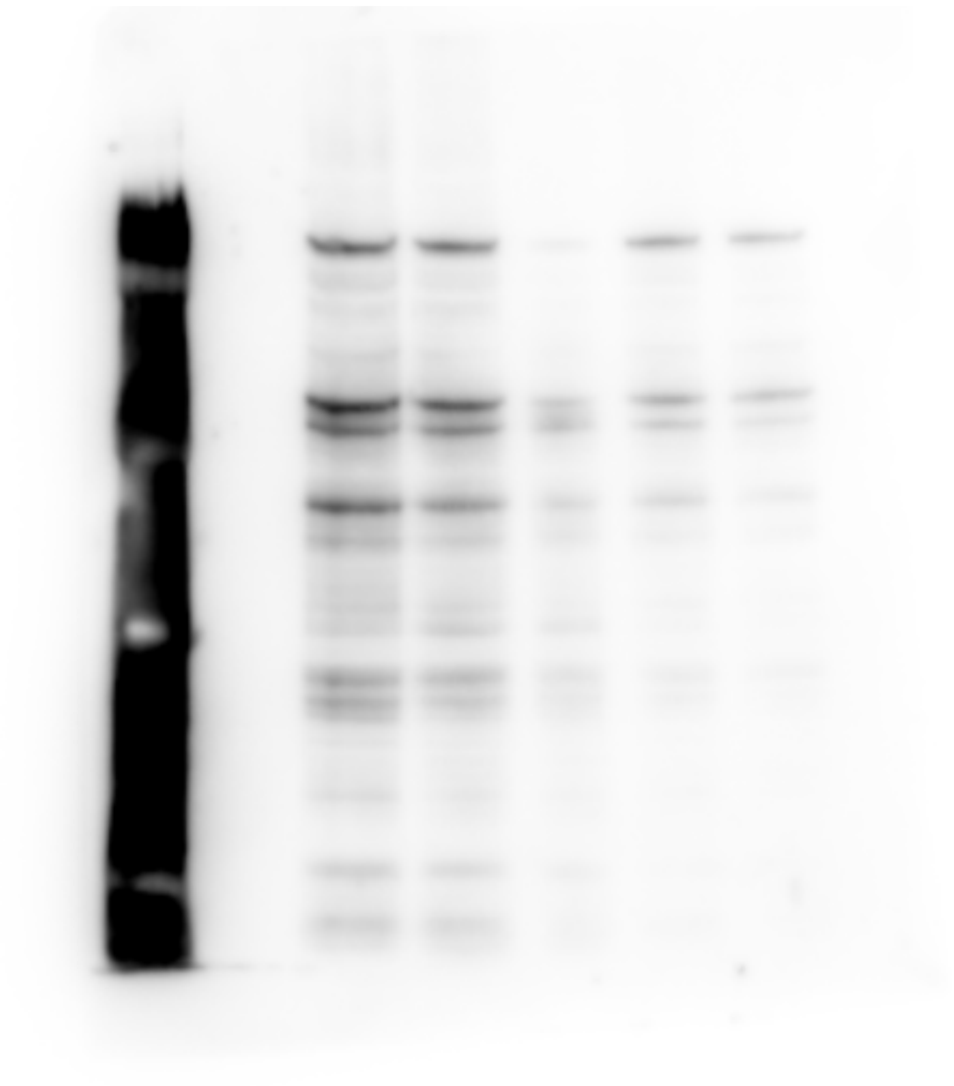

Supplementary Figure 2: A chemiluminescence western blot using wheat germ agglutinin to label whole-cyprid extract, resolved using SDS-PAGE.

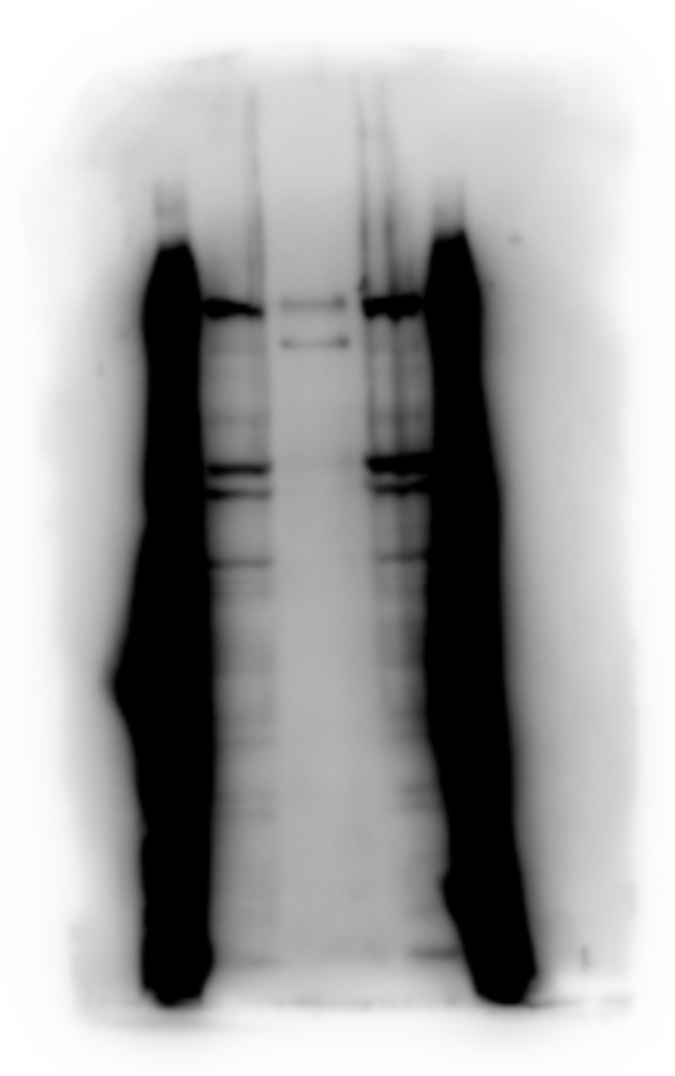

Supplementary Figure 3: A chemiluminescence western blot using wheat germ agglutinin to label cyprid cement glands, resolved using SDS-PAGE.

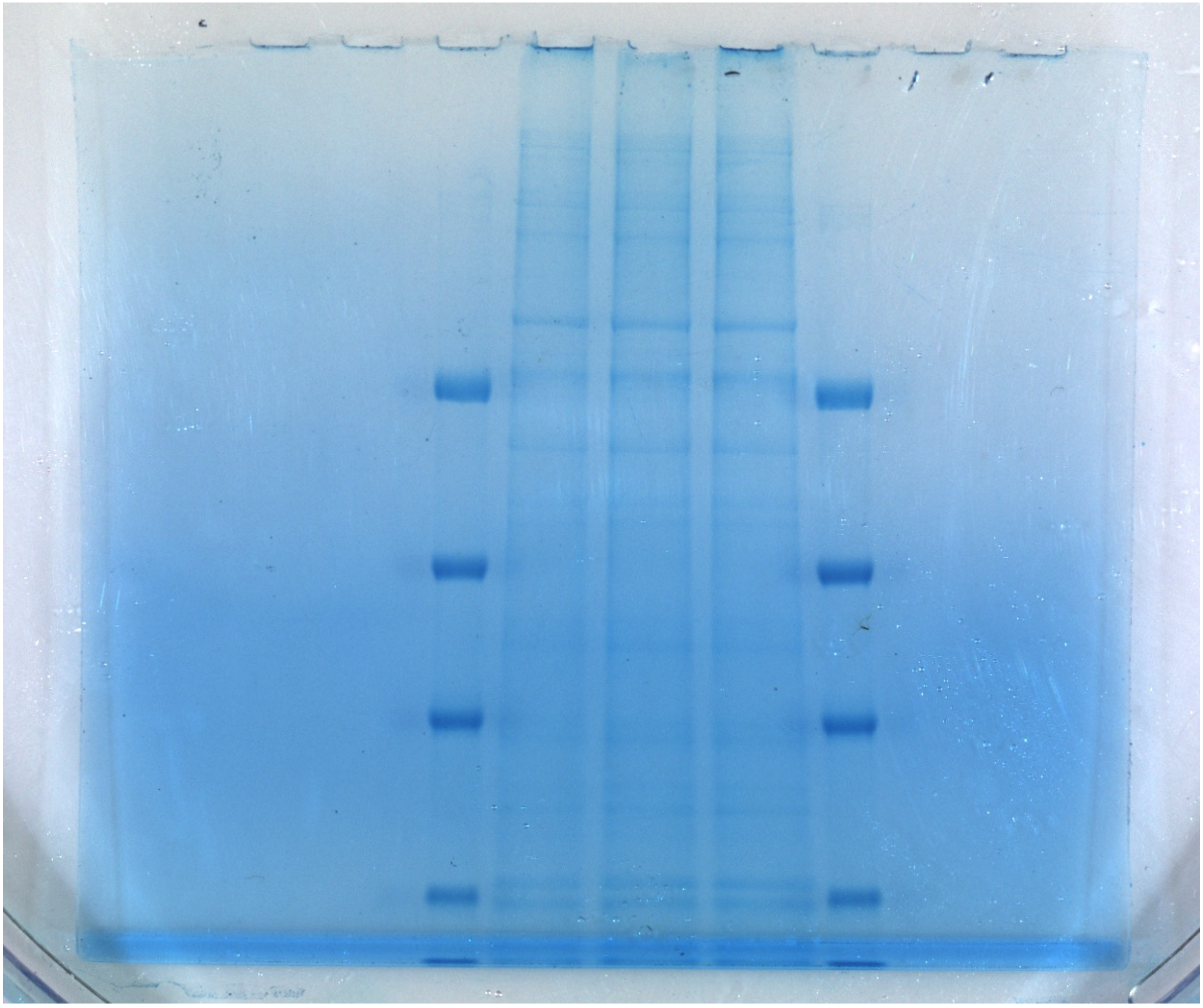

Supplementary Figure 4: An SDS-PAGE gel of whole-cyprid extract immunoprecipitated using wheat germ agglutinin.

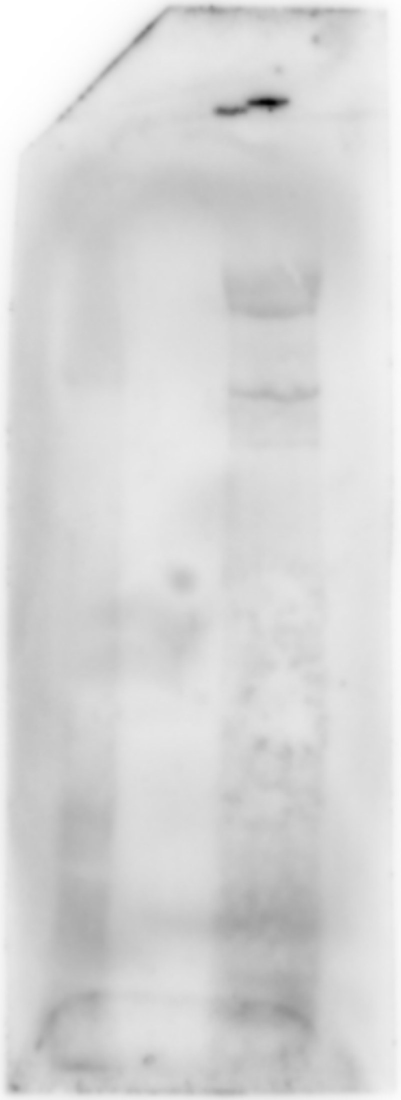

Supplementary Figure 5: A chemiluminescence western blot using the chitin-binding domain to label whole-cyprid extract, resolved using SDS-PAGE.

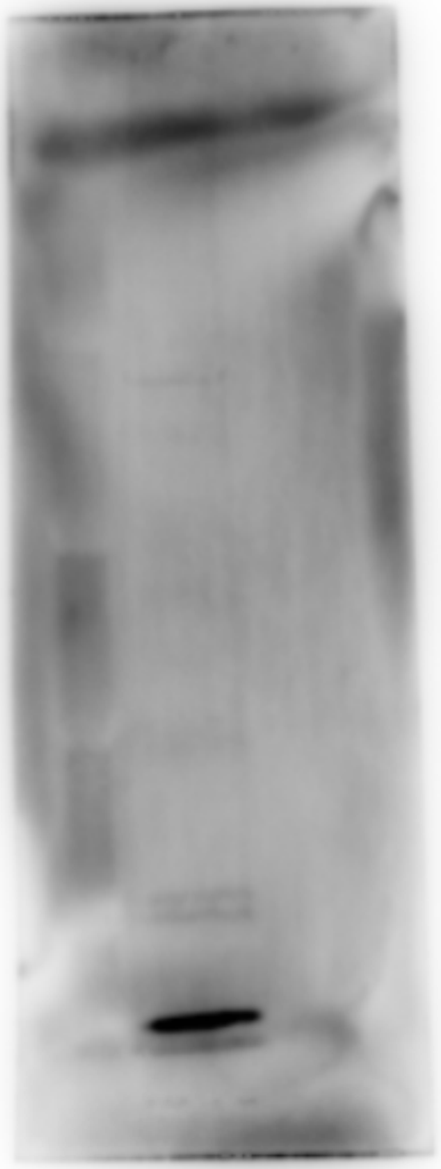

Supplementary Figure 6: A chemiluminescence western blot using an antibody to Mr-lcp1-122k to label whole-cyprid extract, resolved using SDS-PAGE.

Supplementary Table 1: A summary of MS/MS data (Supplementary Data 1 & Supplementary Table 2), produced from WGA-immunoprecipitated cyprid proteins, used to identify 4 contigs from the *B. amphitrite* transcriptome that matched the Mr-lcp1-122k cement gland-specific protein in *M. rosa*.

**Representative *B. amphitrite* contig**	**Identity with equivalent *M. rosa* 122 kDa sequence**	**No. related contigs matched by MS/MS**	**No. peptides per related contig**	**Representative contig (column 1) coverage by MS/MS peptides (column 4)**
k55_2957215	72 %	4	4;2;2;1	59 %
k75_2477949	65 %	3	3;3;3	34 %
k75_2510032	66 %	3	3;3;2	29 %
K55_979678	69 %	3	3;3;2	20 %

Supplementary Table 2: Amino acid sequence fragments identified by Edman degradation of gel bands, relating to Mr-lcp1-122k.

Band1-PS1	K or R / xLPsNTVstiqqa
Band1-PS2	K or R / LTPGILQAAr
Band1-PS3	K or R / xxglv
Band1-PS4	K or R / AFQPVPYLQY-
Band1-PS5	K or R / FsLTDnef-
