# Supplementary material for: Chitin is a functional component of the larval adhesive of barnacles
Source: Commun Biol. 2020 Jan 17;3:31. doi: 10.1038/s42003-020-0751-5 (PMC6969031; doi:10.1038/s42003-020-0751-5)
Supplement: Supplementary file 2 — Description of Additional Supplementary Files [file 42003_2020_751_MOESM2_ESM.docx]

**Supplementary Data (stand-alone Excel files)**

**Supplementary Data 1:** MS/MS generated peptides from two SDS-PAGE bands ('upper' and 'lower'), searched against the *B. amphitrite* transcriptome (>200 nucleotide cutoff) using MaxQuant. ID requirements included >7 amino acids matched per peptide and >2 peptides per matched contig.

**Supplementary Data 2:** The results of an experiment to determine the effects of chitinase exposure on the permanent attachment of cyprids.
